# Electrocardiographic and other Noninvasive Hemodynamic Markers in Decompensated CHF Patients

**DOI:** 10.3390/jcdd10030125

**Published:** 2023-03-15

**Authors:** Gianfranco Piccirillo, Federica Moscucci, Martina Mezzadri, Cristina Caltabiano, Ilaria Di Diego, Myriam Carnovale, Andrea Corrao, Sara Stefano, Claudia Scinicariello, Marco Giuffrè, Valerio De Santis, Susanna Sciomer, Pietro Rossi, Damiano Magrì

**Affiliations:** 1 Department of Clinical and Internal Medicine and Anesthsiological and Cardiovascular Sciences, Policlinico Umberto I, “La Sapienza” University of Rome, 00185 Rome, Italy; 2 Department of Internal Medicine and Medical Specialties, Policlinico Umberto I, “La Sapienza” University of Rome, 00185 Rome, Italy; 3 Cardiology Division, Arrhytmology Unit, S. Giovanni Calibita Hospital, 00186 Rome, Italy; 4 Dipartimento di Medicina Clinica e Molecolare, S. Andrea Hospital, “Sapienza” University of Rome, 00185 Rome, Italy

**Keywords:** acutely decompensated chronic heart failure, intrinsicoid deflection time, ECG markers, tele-monitoring, prevention

## Abstract

Acutely decompensated chronic heart failure (adCHF) is among the most important causes of in-hospital mortality. R-wave peak time (R_p_T) or delayed intrinsicoid deflection was proposed as a risk marker of sudden cardiac death and heart failure decompensation. Authors want to verify if QR interval or R_p_T, obtained from 12-lead standard ECG and during 5-min ECG recordings (II lead), could be useful to identify adCHF. At hospital admission, patients underwent 5-min ECG recordings, obtaining mean and standard deviation (_SD_) of the following ECG intervals: QR, QRS, QT, JT, and T peak–T end (Te). The R_p_T from a standard ECG was calculated. Patients were grouped by the age-stratified Januzzi NT-proBNP cut-off. A total of 140 patients with suspected adCHF were enrolled: 87 (mean age 83 ± 10, M/F 38/49) with and 53 (mean age: 83 ± 9, M/F: 23/30) without adCHF. V_5-_, V_6-_ (*p* < 0.05) R_p_T, and QR_SD_, QRS_SD_, QT_SD_, JT_SD_, and Te_SD_ *p* < 0.001 were significantly higher in the adCHF group. Multivariable logistic regression analysis demonstrated that the mean of QT (*p* < 0.05) and Te (*p* < 0.05) were the most reliable markers of in-hospital mortality. V_6_ R_p_T was directly related to NT-proBNP (r: 0.26, *p* < 0.001) and inversely related to a left ventricular ejection fraction (r: 0.38, *p* < 0.001). The intrinsicoid deflection time (obtained from V_5-6_ and QR_SD_) could be used as a possible marker of adCHF.

## 1. Introduction

Chronic heart failure (CHF) is still a serious, unresolved problem that undermines the economic sustainability of all national healthcare systems [1,2]. The massive hospital admission rate for acute, decompensated CHF (dCHF) poses a major burden on healthcare systems, being costly and negatively impacting patients’ quality of life. Obviously, the current COVID-19 pandemic has dramatically challenged and stressed all health systems, worsening the already critical situation of chronic patients, increasing hospital admissions as well as mortality and morbidity [3,4,5,6]. Thus, the remote monitoring of CHF patients seems to represent the most easy and cost-effective solution, utilized in order to adapt and customize drug therapy to prevent new decompensations and hospitalizations [6]. Then, the identification of new non-invasive, low-cost, simple, and transmissible markers to monitor these patients seems mandatory. Recently, some ECG markers able to identify adCHF patients have been studied; in particular, markers of short-term repolarization variability appeared promising to individuate patients with high levels of NT-proBNP [7,8], which is the gold standard for the diagnosis of acute CHF patients [9] and high mortality risk [7,8,10,11,12]. In addition, it was reported that an increase in the R-wave peak time (R_p_T) or, more specifically, in the intrinsicoid deflection time (R_p_T obtained only in the precordial unipolar leads) [13] was a risk factor for sudden cardiac death [14,15] or heart failure events [16]. The aim of this study was to prove that a simple, inexpensive, non-invasive marker as the intrinsicoid deflection time was suitable for diagnosing adCHF and to verify that short-term RpT variability, expressed as five minutes of mean standard deviation, was consequently affected in these patients.

## 2. Materials and Methods

A total of 140 consecutive patients were enrolled at the admission to our Geriatric Department from January 2019 to July 2022 due to dyspnea or other symptoms of decompensated chronic heart failure (CHF), diagnosed according to the 2021 European Society of Cardiology heart failure guideline [9]. Patients have been grouped in two different groups (subjects with or without adCHF) according to the age-stratified value of the NT-proBNP cut-off proposed by Januzzi [17]. In particular, the cut-offs were 450, 900, and 1800 pg/mL, respectively, for patients less than 50, between 50 and 75, and older than 75 years old [17]. Exclusion criteria were: complete left or right ventricular branch block, intra-ventricular conduction delay (120 ms), or paced QRS; and active pneumonia, SARS-CoV2 infection, or high degree of cognitive impairment which leaves the patients unable to sign the written informed consent for the participation in the study. All patients had stable previous clinical conditions at home with NYHA class II–III, and all of them were in the IV NYHA functional class at the time of enrollment. At the study run-in, each patient underwent a full clinical history, physical examination, standard ECG, transthoracic echocardiogram, and simultaneously 5 min of noninvasive hemodynamic evaluation using bioimpedance cardiography (Physioflow, Manatec Biomedical, Folschviller, France) and a single-lead (II lead) ECG recording. Furthermore, at this time, a blood sample for NT-proBNP dosage (Alere Triage Analyzer, Alere, San Diego, CA, USA) as well other serum variables was collected. ECG signals were acquired and digitalized with a custom-designed card (National Instruments, Austin, TX, USA) at a sampling frequency of 500 Hz. Measurements used for the ECG segment and interval analysis were detected automatically using a classic adaptive first derivative/threshold algorithm and template method [18]. Our research group designed and produced software for data acquisition, storage, and analysis with the LabView program (National Instruments, Austin, TX, USA). An expert cardiologist (GP) checked the different ECG intervals and segments, automatically marked using the software, and, when needed, manually corrected the mistakes [18,19,20,21,22]. The mean and standard deviations (_SD_) of the following study intervals and segments on five minutes ECG recordings were recorded: mean of R–R intervals (RR); the mean of Q–R intervals (QR): from q to the peak of R waves (R-wave peak time); the mean of Q–R–S intervals (QRS): from q to S waves; the mean of Q–T intervals (QT): from q to end of T waves; and the mean of ST end segments (ST) from S to the end of T waves. RR_SD_ was reported only in sinus rhythm subjects and without premature supraventricular or ventricular beats. In patients with a sinus rhythm, a power spectral analysis with an autoregressive algorithm was also used and evaluated [8,23,24,25,26,27]. Next, the total area under the spectra was defined as the total power (TP) and it was equivalent to the variance in the RR; very low-frequency power (VLF) was the area between 0 and 0.04-hertz equivalents (Hz); low-frequency power, those between 0.04 and 0.15 (Hz); and high-frequency power (HF), those between 0.15 and 0.40 hertz. We also calculated the ratio between LF and HF (LF/HF). In addition, we transformed the absolute power in the LF and HF normalized units (nu) [23,24,25,26,27,28]. The R_p_T was calculated manually using an electronic caliper on a 12-lead ECG (Esaote, model: MyCardioPad/XL 12 channels, Florence, Italy) available for the analysis (paper speed was 25 mm/s and calibration 10 mm/mV). Inter-observer measurement error was avoided by using measurements made by the same trained operator. The intra-observer and measurement errors of R_p_T were defined.

Bioimpedance cardiography was used to obtain non-invasive hemodynamic data. In particular, patients underwent 5-min recordings to obtain heart rate (b/m), stroke volume (SV) (mL), stroke volume index (SVI) (mL·m^2^), cardiac index (CI) (L/m·m^2^), systemic vascular resistance index (SVRI) (Dyn.s/cm^2^·m^2^), left ventricular end-diastolic volume (LVEDV) (mL), left ventricular end-systolic volume (LVESV = LVEDV-SV) (mL), contractility index (ConI), left ventricular ejection time (LVET) (ms), left cardiac work index (LCWI) (kg.m·m^2^), early diastolic filling ratio (EDFR), and left ventricular ejection fraction (LVEF_Bio_) [29,30,31,32,33,34,35].

## 3. Statistical Analysis

Data were reported as mean ± standard deviations or as median and interquartile ranges for normal and skewed distribution data. The categorical variables were reported as frequencies and percentages. We compared non-normally (as evaluated using the Kolmogorov–Smirnov test) and normally distributed data, respectively, using the Mann–Whitney test and unpaired *t*-test. Categorical variables were compared using the χ^2^ test. Uni- and multi-variable forward (A. Wald) stepwise logistic regression analyses were used to determine the association between mortality and the studied variables (QT and Te intervals). Stepwise multiple regression analysis was used to determine possible relationships between the studied variables. Receiver operating characteristic (ROC) curves were used to determine the sensitivity and specificity of the studied parameters predictive of decompensated CHF (positive NT-proBNP with Januzzi cut-off) and areas under the curves (AUCs), and 95% confidence intervals (CI) were calculated to compare the diagnostic accuracy. For statistical analysis, we used SPSS-PC+ (SPSS-PC+ Inc., Chicago, IL, USA). We considered statistically significant *p* values < 0.05.

## 4. Results

217 patients were eligible, 23 patients were not included because the quality of standard ECG or short-term ECG recordings was not optimal for the study, and 54 patients were not included for intra-ventricular conduction delay or complete left or right branch block. Therefore, we conducted the study on 140 patients (Table 1) regarding R_p_T and short-term ECG recording data; at the same time, we obtained hemodynamic data only from 58 subjects due to the availability of the necessary tool (from January 2021).

According to the NT-proBNP Januzzi cut-off, 87 patients were grouped as adCHF and 53 as patients without decompensated CHF. During the hospitalization, a total of thirty-two patients died (overall mortality rate, 23%), nineteen (14%) died for bronchopneumonia and respiratory failure (one patient for COVID-19 pneumonia), nine for terminal heart failure (6%), two for fatal myocardial infarction (1%), two for sudden cardiac death (one for sustained ventricular tachycardia and ventricular fibrillation; one for acute cor pulmonale secondary to massive embolism). The overall cardiovascular mortality rate was 9%. Regarding the deceased patients, 29 out of 32 (91%) were in decompensated CHF group (*p* < 0.001) (Table 1).

A echocardiographic left ventricular ejection fraction (LVEF_Echo_) (*p* < 0.001) and creatinine clearance (*p* < 0.05) were significantly lower in patients with decompensated CHF (Table 1) in comparison to the patients without. As expected, NT-proBNP (*p* < 0.001), high sensitivity troponin (*p* < 0.001), and a tricuspidal regurgitation peak gradient (*p* < 0.05) were significantly higher in patients with adCHF. Moreover, adCHF patients reported a higher fasting glucose (*p* < 0.05), but they presented a lower total (*p* < 0.05), HDL (*p* < 0.05), and LDL (*p* < 0.05) cholesterol.

Patients with adCHF showed heart failure, with a more reduced ejection fraction (*p* < 0.05) (Table 1); among patients without adCHF, CHF with a preserved ejection fraction was highly represented (*p* < 0.05) (Table 1). Finally, renal insufficiency and permanent atrial fibrillation significantly prevailed in the adCHF group. This last group showed a higher level of patients treated with furosemide (*p* < 0.05), β-blockers (*p* < 0.05), and aldosterone antagonists (*p* < 0.05). On the contrary, patients without adCHF reported a more prevalent use of ACE/sartans (*p* < 0.05) (Table 1). The delayed intrisicoid deflection (V_5_-V_6_) being higher than 50 ms was the same in both study groups (Table 1).

Subjects with adCHF showed a significantly longer R_p_T in V5 (*p* < 0.05), V6 (*p* < 0.05) (intrinsicoid deflection time), and maximum R_p_T (*p* < 0.05) in comparison to the group without decompensated CHF (Table 2). Intra-observer variability was different for each lead, but never exceeded the value of 3 ms.

Subjects with adCHF showed a shorter RR (*p* < 0.05) and a longer Te (*p* < 0.05) in comparison to the group without decompensated CHF (Table 2). In addition, QR_SD_ (*p* < 0.001), QRS_SD_ (*p* < 0.001), QT_SD_ (*p* < 0.001), ST_SD_ (*p* < 0.001), and Te_SD_ (*p* < 0.001) were significantly higher in decompensated patients than in the others (Table 3).

The adCHF subjects had lower RR_SD_ (*p* < 0.05), TP (*p* < 0.05), VLF (*p* < 0.05), LF (*p* < 0.05), and HF (*p* < 0.05) (Table 4).

β-blockers and calcium antagonists did not have any influence on the R_p_T; in fact, even comparing the groups for treatments, no significant difference between patients with or without antiarrhythmic drugs was found.

Univariable logistic regression analysis reported a strong relationship between in-hospital mortality and QT (odds ratio: 1.02, 95% confidence limit: 1.00–1.03, *p* < 0.05), Te (odds ratio 0.93, 95% confidence limit: 0.89–0.97 (*p* < 0.05), and Te_SD_ (odds ratio: 0.90, 95% confidence limit: 0.82–0.98, *p* < 0.05). Multivariable logistic regression analysis only selected QT and Te as significant predictors of mortality in hospitalized patients (Table 5).

Regarding the non-invasive hemodynamic data, the adCHF patients reported a lower LVEF_BIO_ (with versus without decompensated CHF subjects: 38 ± 9 vs. 44 ± 9 %, *p* < 0.05) and an increase in the EDFR (with versus without decompensated CHF subjects: 79, i.r. 36 vs. 64, i.r 34, *p* < 0.05).

**Figure 1:**
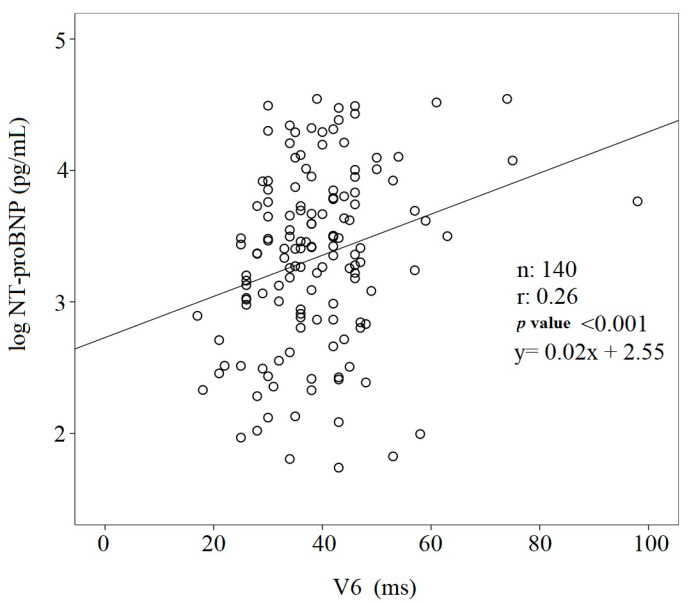
Linear regression between NT-proBNP and V_6_ intrinsicoid deflection time.

**Figure 2:**
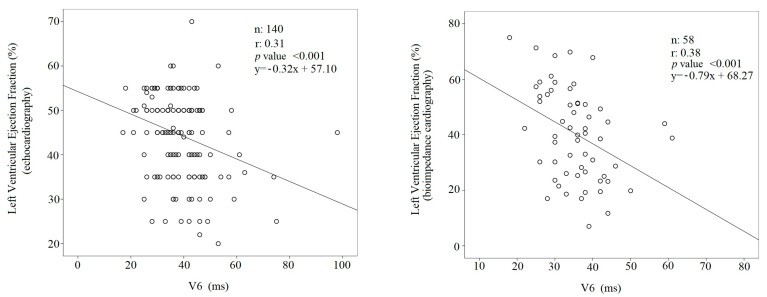
Linear regression between left ventricular ejection fraction and V_6_ intrinsicoid deflection time.

In all study subjects, the V_6_ R_p_T was directly and positively related to the NT-proBNP (r: 0.26, *p* < 0.001) (Figure 1). On the contrary, both LVEFs, namely LVEF_ECHO_ (r: 0.31, *p* < 0.001) and LVEF_BIO_ (r: 0.38, *p* < 0.001), were inversely related to the V_6_ R_p_T (Figure 2).

**Figure 3:**
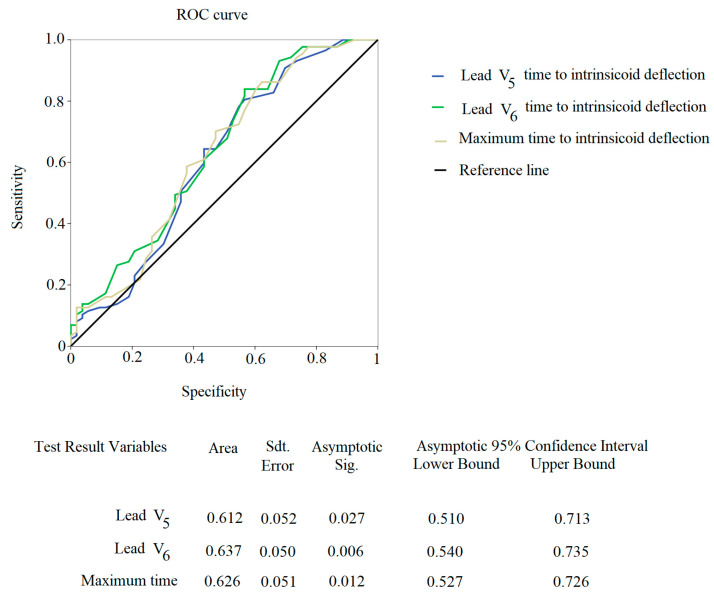
ROC curve of statistically significant unipolar precordial lead (V_5-6_ or maximum time to intrinsicoid deflection) and age-stratified NT-proBNP Januzzi cut-off.

The V_5_ (AUC: 0.612, *p* < 0.05), V_6_ (AUC: 0.637, *p* < 0.05) and maximum (AUC: 0.626, *p* < 0.05) R_p_T showed the ROC curves with the highest sensitivity/specificity for positive Januzzi NT-proBNP levels for adCHF (Figure 3).

**Figure 4:**
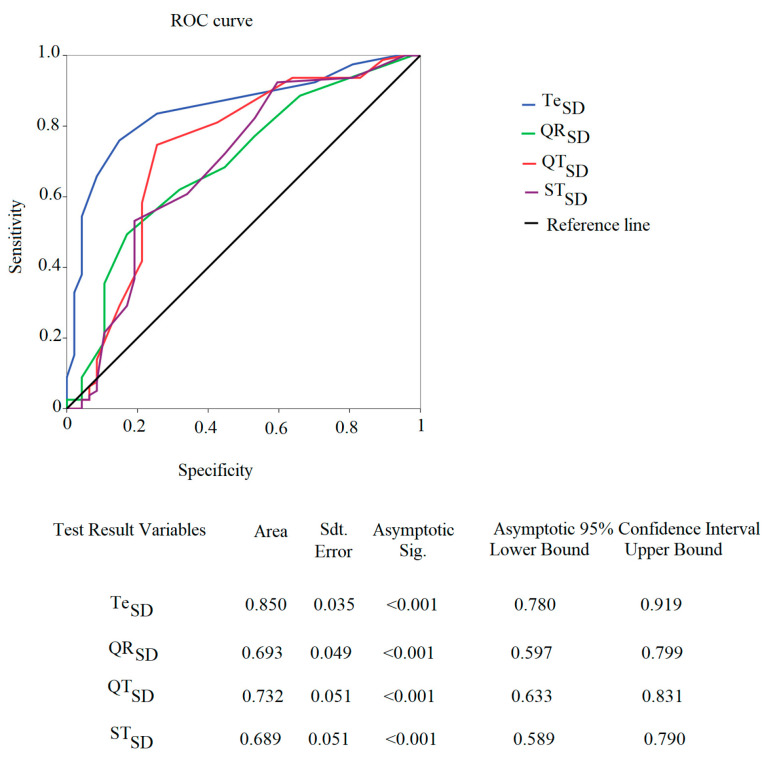
ROC curve of statistically significant standard deviations (SD) of Te, QR, QT, and ST and age-stratified NT-proBNP Januzzi cut-off.

QR_SD_ (AUC: 0.693, *p* < 0.001), QT_SD_ (AUC: 0.732, *p* < 0.05), ST_SD_ (AUC: 0.689, *p* < 0.05), and Te_SD_ (AUC: 0.850, *p* < 0.05) showed significant ROC curves for positive Januzzi NT-proBNP levels for adCHF (Figure 4). No other variables showed differences in the studied groups.

## 5. Discussion

The major findings of this study were that the intrinsicoid deflection time, obtained from the unipolar precordial leads (V_5-6_) of a standard ECG, was higher in patients with adCHF. Secondly, these patients showed an increased QR_SD_; in fact, we observed a higher level of R_p_T in short-term temporal dispersion, obtained in lead II, in patients with adCHF. To avoid misunderstandings, it is important to reiterate the definition of R_p_T and intrinsicoid deflection time: the first one regards the interval between q and R waves in all ECG leads, the second one represents the same ECG interval but obtained in the precordial leads. Moreover, the delayed intrinsicoid deflection lasts equal to or more than 50 ms in the V_5_ and V_6_ leads [13,36]. Normally, this interval lasts less than 50 ms in healthy subjects, but it tends to grow with the increase in cardiac hypertrophy [36], and it was proposed as a risk marker of sudden cardiac [14,15] or heart failure acute events [16]. Our data showed that this interval is affected by the ejection fraction more than by the left ventricular hypertrophy degree; so, it could be considered as a left ventricular function marker rather than a heart hypertrophy marker. This finding confirmed that this electrical marker could be used to monitor CHF patients.

From an electrophysiological point of view, the R_p_T coincides with the upstroke velocity (phase 0) of the ventricular myocyte action potential, and it is characterized by the rapid inflow of sodium ions in the ventricular myocardiocytes. Normally, the duration of this phase is of a few milliseconds, but in dilative or hypertrophic myocardiopathy, it is associated with electrical myocardial remodeling, an increase in action potential duration and its temporal inhomogeneity, as well as with an increase in R_p_T and intrinsicoid deflection velocity. The electrophysiological basis, studied in different animal models, could involve the sodium, calcium, and potassium channels, but also structural changes such as the reduction in gap junctions, fibrosis, muscle fiber disarray, and cellular size [36]. Therefore, multifactorial mechanisms could be involved in RpT prolongation, but the neurohumoral activation in CHF decompensated patients could probably play the lead role. High catecholamine levels, inducing a downregulation of β-adrenergic myocardial receptors, could reduce chrono/dromo/inotropy, causing an increase in the R_p_T too. In the present study, we observed a decrease in all spectral components and total short-term variability, expressed as both TP and RR_SD_; this behavior was observed in advanced CHF, in the animal experimental model [19,26], and the clinical study, too [24,27,37,38,39,40,41].

Another interesting point was that, despite the drug therapy with potential effects (β-blockers, calcium channel blockers, and antiarrhythmics) on the R_p_T in these patients, no possible influence of therapy on the studied patients was detected, probably due to the receptors’ downregulation, frequently seen in these patients; therefore, these findings confirmed the handling of intrinsicoid deflection and the short period of R_p_T temporal dispersion as markers of heart failure decompensation. Finally, an increase in the short period temporal dispersion of different ECG intervals or segments was predictive of CHF decompensation, as previously observed [7,8]. On the contrary, the possible use of delayed intrinsicoid deflection or R_p_T as a sudden cardiac death marker in these patients was not confirmed, as our study reported only two cases of sudden cardiac death.

Finally, the bioimpedance analysis was useful to individuate patients with adCHF, specifically thanks to two parameters: LVEF_BIO_ and EDFR. The first was indirectly calculated according to the Capan equation [42], and the second was an index of diastolic dysfunction. In fact, this last index was positively related to the peak Doppler echocardiographic early diastolic velocity [43]. In conclusion, these non-invasive hemodynamic indexes could find a prominent place in the possible remote monitoring of these patients, but new prospective studies are needed.

## 6. Conclusions

The continuous aging of the population and the recent pandemic have increasingly highlighted the need to promote health and quality of life, especially for patients with chronic diseases. Heart failure, especially in its more advanced stages, is subject to frequent exacerbations that put patients’ lives at risk and weigh heavily on health systems. The phenomenon of the “revolving door” in fact determines the increasingly frequent need for hospitalization due to the rapid succession of acute decompensation events. The possibility of remotely monitoring chronic patients should be applied to all subjects with chronic heart failure, in order to guide therapeutic choices and avoid exacerbations. Non-invasive technologies specifically suited for elderly and frail patients with CHF are not yet widely available. In this study, we attempted to evaluate adCHF using simple, inexpensive, easily interpretable, and repeatable electrical markers. The hope is to improve the monitoring and therapy of outpatients with CHF and reduce the high rate of rehospitalization. Present and future research should point towards this new frontier and beyond the challenges of the coming years.

## 7. Limitations

A current limitation of the present study is represented by the paucity of patients, despite the sample size having been statistically calculated and allowing for a reliable statistical significance of the results. It will be desirable to plan a multicenter study with a larger case series to confirm the data obtained.

Furthermore, the enrolled population is basically composed by elderly patients. This aspect is therefore not attributable to an arbitrary choice made by the authors, but to the characteristics of the population under study. In fact, heart failure in the advanced stage with frequent episodes of decompensation is prevalent in the elderly population; so, the mean age of both the heart failure group and the control group remains above 80 years. In our opinion, this aspect does not limit the results of the study; on the contrary, it highlights a positive trend in the data obtained which, while requiring further studies, lay the foundations for the use of these clinical and electrocardiographic parameters in telemedicine and telemonitoring, specifically useful in this category of fragile patients. A separation into groups by age was followed due to the existence of different cut offs of NT-proBNP depending on the age of the patients according to the Januzzi criteria [17]. The authors do not exclude that with other classifications, it would be possible to obtain other results. At the moment, however, it seems logical to follow this type of subdivision.

Another point is that the intrinsicoid deflection may be subject to numerous “disturbing elements” and biases; nevertheless, we did not observe substantial differences among the patients in the study and the control group (i.e., drugs intake). These data therefore reinforce the observation and the diagnostic and prognostic power of the variables studied.

In conclusion, the data obtained in this study, although preliminary, are in line with the existing literature, confirming the possibility of creating a multiparametric score that is easy and immediate to use and rather cheap, all in order to stage the risk of acute decompensation or the mortality of patients affected by decompensation.

## Figures and Tables

**Table 1:** General characteristics of the study subjects.

	Subjects with	Subjects without	
	Acute Decompensated Heart Failure		
	N: 87	N: 53	*p* value
**Age, years**	83 ± 10	83 ± 9	0.884
**M/F n**	38/49	23/30	0.974
**BMI, kg/m^2^**	26 ± 5	25 ± 5	0.814
**Left Ventricular Ejection Fraction %**	42 ± 10	48 ± 8	<0.001
**Left Ventricular Mass Index, (g/m^2^)**	129 ± 33	134 ± 28	0.440
**Left Ventricular End-Diastolic Diameter, (mm)**	53 ± 8	52 ± 6	0.462
**Posterior Wall Thickness, (mm)**	11 ± 2	1 ± 1	0.253
**Interventricular Septum Thickness, (mm)**	12 ± 1	12 ± 1	0.511
**Left Atrial Transversal Diameter, (mm)**	47 ± 6	45 ± 6	0.133
**Tricuspid Annular Plane Systolic Excursion, (mm)**	20 ± 5	21 ± 3	0.194
**Tricuspid Regurgitation Peak Gradient, (mmHg)**	48 ± 15	37 ± 9	0.001
**NT-pro BNP, pg/mL**	4930 {9170}	512 {790}	<0.001
**C-reactive Protein, (mg/dL)**	4 {9}	5 {9}	0.085
**High sensitivity cardiac troponin, (pg/L)**	55 {94}	29 {25}	<0.001
**Serum potassium, (mmol/L)**	4.10 ± 0.64	4.14 ± 0.56	0.716
**Serum Calcium, (mmol/L)**	2.20 ± 0.77	2.10 ± 0.15	0.716
**Creatinine Clearance, (mL/m)**	42 ± 25	54 ± 22	0.005
**Fasting Glucose, (mmol/L)**	7.2 ± 2.5	6.1 ± 1.9	0.011
**HbA_1c_ (%)**	6.3 ± 1.6	5.8 ± 1.2	0.070
**Total Cholesterol, (mmol/L)**	3.35 ± 0.95	4.00 ± 0.90	0.006
**HDL-cholesterol, (mmol/L)**	0.99 ± 0.39	1.19 ± 0.41	0.030
**LDL-cholesterol, (mmol/L)**	1.67 ± 0.65	2.19 ± 0.74	0.002
**Triglycerides, (mmol/L)**	1.97 ± 1.60	1.50 ± 0.84	0.126
**PaO_2_/FiO_2_ ratio,**	329 ± 123	327 ± 76	0.921
**A-ADO_2_, mmHg**	45 (62)	34 (31)	0.102
**Reduced LVEF, n (%)**	41 (47)	11 (21)	0.002
**Mildly reduced LVEF, n (%)**	14 (16)	8 (15)	0.875
**Preserved LVEF, n (%)**	32 (37)	34 (64)	0.002
**Hypertension, n (%)**	65 (75)	42 (79)	0.540
**Hypercholesterolemia. n (%)**	39 (45)	18 (34)	0.204
**Diabetes, n (%)**	40 (46)	16 (30)	0.064
**Renal Insufficiency, n (%)**	48 (55)	12 (23)	<0.001
**Known Myocardial Ischemia History, n (%)**	33 (38)	13 (25)	0.102
**Valve Diseases,**	25 (29)	13 (25)	0.587
**Premature Supraventricular Complexes, n (%)**	8 (9)	2 (4)	0.227
**Premature Ventricular Complexes, n (%)**	20 (23)	7 (13)	0.155
**Permanent Atrial fibrillation, n (%)**	35 (40)	10 (20)	0.009
**Time to Intrisicoid Deflection >50 ms, n(%)**	14 (16)	5 (9)	0.265
**Deceased Subjects, n (%)**	29 (33)	3 (6)	<0.001
**β-blockers, n (%)**	63 (72)	28 (53)	0.018
**Furosemide, n (%)**	72 (83)	33 (62)	0.007
**ACE/Sartans, n(%)**	24 (28)	27 (51)	0.005
**Aldosterone antagonists, n (%)**	15 (17)	3 (6)	0.047
**Potassium, n (%)**	8 (9)	4 (8)	0.735
**Nitrates, n (%)**	12 (14)	9 (17)	0.608
**Digoxin, n (%)**	5 (6)	2 (4)	0.603
**Statins, n (%)**	15 (28)	23 (26)	0.810
**Antiplatelet drugs, n (%)**	28 (32)	22 (42)	0.264
**Oral Anticoagulants, n (%)**	24 (28)	12 (23)	0.516
**Diltiazem or Verapamil, n (%)**	2 (2)	3 (6)	0.299
**Dihydropyridine Calcium channel blockers, n (%)**	11 (13)	12 (23)	0.122
**Propafenone, n (%)**	1 (1)	1 (2)	0.721
**Amiodarone, n (%)**	6 (7)	3 (6)	0.772
**Valsartan/Sacubitril, n (%)**	2 (2)	1 (2)	0.870
**Gliflozin, n (%)**	1 (1)	0 (0)	0.999

M/F: male/female; BMI: body mass index; LVEF: left ventricle ejection fraction. Comparison data between patients with or without decompensated heart failure. Data are expressed as mean± SD, median {interquartile range}, or the number of patients (%).

**Table 2:** R-Peak time in the 12-lead ECG in study subjects.

	Subjects with	Subjects without	
	Acute Decompensated Heart Failure		
	N: 87	N: 53	*p* value
**Lead I, ms**	41 ± 9	41 ± 14	0.951
**Lead II, ms**	42 ± 12	39 ± 9	0.138
**Lead III, ms**	42 ± 12	38 ± 11	0.062
**Lead aVR, ms**	40 ± 9	37 ± 10	0.160
**Lead aVL, ms**	39 ± 10	39 ± 13	0.921
**Lead aVF, ms**	40 ± 9	39 ± 13	0.617
**Lead V_1_, ms**	42 ± 14	38 ± 16	0.124
**Lead V_2_, ms**	41 ± 15	39 ± 14	0.391
**Lead V_3_, ms**	40 ± 13	39 ± 14	0.196
**Lead V_4_, ms**	39 ± 11	36 ± 12	0.112
**Lead V_5_, ms**	40 ± 9	35 ± 11	0.011
**Lead V_6_, ms**	41 ± 11	35 ± 9	0.002
**Maximum R-Peak Time, ms**	42 ± 11	37 ± 10	0.004

Data are expressed as mean ± SD.

**Table 3:** Short-pterm ECG variables in study subjects.

	Subjects with	Subjects without	
	Acute Decompensated Heart Failure		
	N:87	N:53	*p* value
**RR mean, ms**	826 ± 183	887 ± 146	0.039
**QR mean, ms**	40 ± 11	38 ± 11	0.172
**QR_SD_, ms^2^**	5 (4)	3 (4)	<0.001
**QRS mean, ms**	90 ± 18	86 ± 18	0.197
**QRS_SD_, ms^2^**	7 (5)	5 (4)	<0.001
**QT mean, ms**	443 ± 75	425 ± 56	0.171
**QT_SD_, ms^2^**	10 (5)	6 (4)	<0.001
**ST mean, ms**	349 ± 71	337 ± 48	0.301
**ST_SD_, ms^2^**	9 (5)	6 (4)	<0.001
**Te mean, ms**	106 ± 31	96 ± 15	0.021
**Te_SD_, ms^2^**	8 (4)	5 (2)	<0.001

Data are expressed as mean ± SD, median (interquartile range), or number of patients (%).

**Table 4:** Short-term RR power spectral analysis.

	Subjects with	Subjects without	
	Acute Decompensated Heart Failure		
	N: 37	N: 36	*p* value
**RR_SD,_ ms^2^**	16 (9)	23 (19)	0.006
**TP, ms^2^**	242 (292)	506 (1051)	0.007
**VLF, ms^2^**	165 (257)	291 (702)	0.031
**LF, ms^2^**	26 (44)	90 (210)	0.006
**HF, ms^2^**	21 (42)	44 (85)	0.030
**LF CF, Hz**	0.11 ± 0.02	0.09 ± 0.03	0.020
**HF CF, Hz**	0.29 ± 0.10	0.28 ± 0.07	0.465
**LF/HF,**	1.33 (1.53)	1.79 (2.87)	0.214
**LF, nu**	45 (20)	54 (38)	0.151
**HF, nu**	33 (24)	30 (23)	0.282

Data are expressed as mean ± SD, median (interquartile range), or the number of patients (%); TP: total power; VLF: very low-frequency; LF: low-frequency; LF CF: LF central frequency; HF: high-frequency; HF CF: HF central frequency; LF/HF: LF/HF ratio; nu: normalized units.

**Table 5:** Logistic regression analysis between in-hospital mortality (dependent variable) and short-term ECG variables.

Variables	B	Wald	Multivariable Analysis Odd Ratio (95% CI)	*p* Values
QT	0.02	5.97	1.02 (1.00–1.03)	0.015
Te	−0.07	11.09	0.93 (0.89–0.97)	0.001

## Data Availability

Data will be available on explicit request sent to the corresponding author.
